# Study on Volleyball-Movement Pose Recognition Based on Joint Point Sequence

**DOI:** 10.1155/2023/2198495

**Published:** 2023-02-17

**Authors:** Xi Li

**Affiliations:** Physical Education Department, Taihu University of Wuxi, Wuxi 214000, Jiangsu, China

## Abstract

With the high-speed operation of society and the increasing development of modern science, people's quality of life continues to improve. Contemporary people are increasingly concerned about their quality of life, pay attention to body management, and strengthen physical exercise. Volleyball is a sport that is loved by many people. Studying volleyball postures and recognizing and detecting them can provide theoretical guidance and suggestions for people. Besides, when it is applied to competitions, it can also help the judges to make fair and reasonable decisions. At present, pose recognition in ball sports is challenging in action complexity and research data. Meanwhile, the research also has an important application value. Therefore, this article studies human volleyball pose recognition by combining the analysis and summary of the existing human pose recognition studies based on joint point sequences and long short-term memory (LSTM). This article proposes a data preprocessing method based on the angle and relative distance feature enhancement and a ball-motion pose recognition model based on LSTM-Attention. The experimental results show that the data preprocessing method proposed here can further improve the accuracy of gesture recognition. For example, the joint point coordinate information of the coordinate system transformation significantly improves the recognition accuracy of the five ball-motion poses by at least 0.01. In addition, it is concluded that the LSTM-attention recognition model is not only scientific in structure design but also has considerable competitiveness in gesture recognition performance.

## 1. Introduction

The posture of the human body is one of the important biological characteristics of the human body. It has many application scenarios, such as gait analysis, video surveillance, augmented reality, human-computer interaction, finance, mobile payment, entertainment and games, and sports science. Gesture recognition allows computers to know what a person is doing and who they are. Especially in the field of monitoring, it is a good solution when the resolution of the face image obtained by the camera is too small. It can also be used as an important auxiliary verification method in the target identification system to reduce the effect of misidentification. Human gesture recognition includes action recognition and identity recognition, and the key lies in human feature extraction. The human body feature extraction mainly completes action feature extraction and identity feature extraction. In volleyball, detecting and identifying relevant poses in motion sequences can not only provide coaches and players with data-based guidance and advice but also help referees make fair and reasonable decisions in various games. However, most of the existing human gesture recognition methods are aimed at the recognition of daily simple actions [1]. Data collection is simple and further research is needed.

Most of the traditional human pose recognition research is done on sequences of video frames [2]. Although some research results have been achieved, it is difficult to break through the bottleneck of human pose recognition research using video due to changes in light intensity, interference from complex backgrounds, and the self-occlusion of target users. In the recent years, with the rapid development of video capture technology, such as Kinect, researchers can easily obtain coordinated information on the image, depth image, and skeletal joint points [3, 4]. The information provided by depth images [5] can reflect the three-dimensional structural information and the geometry of target objects well compared with the images. Moreover, it has strong robustness to the influence of factors such as light intensity and scale changes. The posture of ball sports is more complex compared with the simple daily posture of the human body, and it also has requirements for research data. Besides, the existing recognition methods cannot effectively judge the ball movement gestures due to the change in the difficulty of gesture recognition. Therefore, further research on pose data and recognition methods is needed to improve the accuracy of ball-motion pose prediction classes.

In this article, volleyball is represented. The problem of volleyball-gesture recognition is studied combined with the analysis and summary of the existing research on human gesture recognition based on the joint point sequence and the long short-term memory (LSTM) network [6]. In addition, this article proposes a data preprocessing method based on the angle and relative distance feature enhancement and a volleyball-motion pose recognition model based on LSTM-attention. Experimental results show that the data preprocessing method reported here can further improve the accuracy of gesture recognition. Besides, the LSTM-attention recognition model is not only scientific in structure design but also has considerable competitiveness in gesture recognition performance, which can provide a basis for the research on gesture recognition in volleyball. Although the LSTM-attention recognition model is not only scientifically designed in structure but also quite competitive in gesture recognition performance, this approach is not yet universal and should be refined in future studies.

## 2. Materials and Methods

### 2.1. Recurrent Neural Network (RNN)

**Figure 1:**
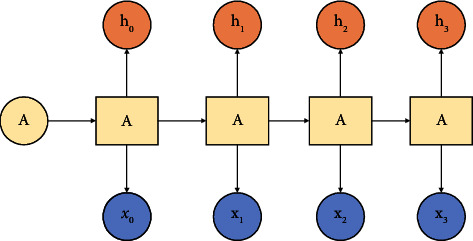
RNN structure diagram.

The RNN is obtained by simulating the human neural transmission system [7]. When humans are thinking, such as reading an article, they may not be able to understand the meaning of the article only by relying on the information currently read. It is often necessary to combine the previous content to understand the essence of the article. This shows that humans are not in a blank brain when they think about problems. The brain will not discard the content of the articles that have been read before but will understand and analyse based on the previous readings. This reveals that human thinking is a continuous process [8]. However, traditional neural networks cannot achieve this, so there is an RNN. The RNN is a neural network with a short-term memory and continuously transmits information by adding loops to the network. It is suitable for processing sequential data.  Figure 1 shows the general structure of an RNN. In Figure 1, *h*_*t*_ is the value of the hidden layer at time *t*, *x*_*t*_ is the input of the network at time *t*, and *h*_*t*_ is its output.

**Figure 2:**
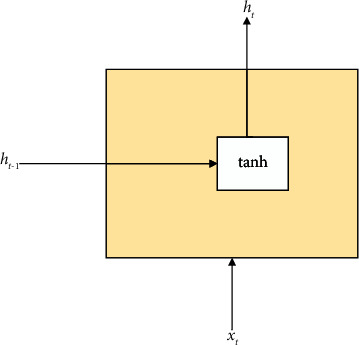
RNN internal structure diagram.

Each block of the neural network in Figure 1 is represented by *A*.  Figure 2 shows the extended structure of the RNN, which is also the internal structure of the RNN transformed according to the time dimension [9]. In the RNN, there is a signal transfer between all the hidden layer nodes [10]. The output of the hidden layer of the RNN at time *t* is fed back to the RNN at time *t *+* *1. Then, the input of the RNN at time *t *+* *1 and the output of the RNN at time *t* will act on the output of the RNN at time *t *+* *1. The chain structure of the RNN essentially determines its strong processing capability for the sequence data. In the recent years, the RNN has performed very well in language modeling, speech recognition, image captioning, and machine translation.

The number of units in the input layer of a neural network is fixed, so inputs of variable length must be processed in a loop or recursion. RNN implements the former. It works by dividing an input of variable length into small chunks of equal length. Then, they are sequentially input into the network, realizing the processing of the variable length input by the neural network. The RNN can encode a tree/graph structure information as a vector, mapping the information into a semantic vector space. This semantic vector space satisfies a class of properties. For example, semantically similar vectors are close together. However, a big limitation of the RNN is the vanishing gradient problem [11]. The RNN is a short-term memory neural network that can only memorize short-distance information sequences. When the time interval becomes large, the RNN will gradually lose its ability to learn the information of the previous time nodes, which will make the learning of the RNN very difficult. This problem is also known as the “long sentence dependency problem.”

### 2.2. LSTM

The researchers make related improvements to its basic structure to solve the “long sentence dependency problem” of the RNN. The researchers design the cell state internally to record the historical state information [12] and introduce the gating unit to control the node information of the hidden layer. This variant of the RNN can solve the above problems very well. It can memorize long-term information related to the current recognition task. This variant is called the LSTM network.

LSTM can be regarded as a special type of the RNN [13], which can greatly enhance the network's ability to store information within long time intervals. For LSTM and the RNN, the same is that they are both chain structures, and the difference is the structure inside their network. LSTM is the most effective sequence model in deep learning (DL) [14], which mainly consists of the forget gate, input gate, and output gate. The RNN model has the problem of missing gradients. LSTM effectively avoids this drawback and proposes a new cell structure, which can judge the retention or forgetting of data. Real-time data are processed from the far left to the far right [15]. Also, the data are processed from the input. Therefore, it is necessary to judge which information continues to run and which is abandoned in the endless input information. This process follows a switch control, which is *f*^((*t*)).

The control function is as follows:

(1) $$\begin{array}{c} f^{\left(t\right)}=\sigma \left(w_{f}\left[h^{\left(t-1\right)},x^{t}\right]+b_{f}\right)\text{.} \end{array}$$

In equation (1), *w*_*f*_ and *b*_*f*_ are the weight and bias of the forget gate, respectively. The previous information is input into the input gate. The task at this layer is to decide which information needs to be updated and how much to update. *σ* represents the activation function, *b*_*f*_ represents the bias value, *h*^(*t* − 1)^ represents the short-term memory, and *x*^*t*^ represents the current input.

(2) $$\begin{array}{c} i^{t}=\sigma \left(w_{i}\left[h^{\left(t-1\right)},x^{t}\right]+b_{i}\right)\text{,} \end{array}$$

(3) $$\begin{array}{c} c^{t}=\sigma \left(w_{c}\left[h^{\left(t-1\right)},x^{t}\right]+b_{c}\right), \end{array}$$

(4) $$\begin{array}{c} C^{t}=i^{t}*c^{t}+f^{\left(t\right)}*C^{t-1}. \end{array}$$

In equations (2)–(4), *w*_*i*_ and *w*_*c*_ represent the corresponding weights, *b*_*i*_ and *b*_*c*_ represent the corresponding biases, and *C*^*t*^ represents the current cell state value. After the screening of the first two gates is completed, the output gate determines which information needs to be output. There is a switch to control the output in the output gate.

(5) $$\begin{array}{c} \sigma^{t}=\sigma \left(w_{o}\left[h^{\left(t-1\right)},x^{t}\right]+b_{o}\right)\text{,} \end{array}$$

(6) $$\begin{array}{c} h^{t}=o^{t_{*}}\;\tanh^{-1}\left(c^{t}\right)\text{.} \end{array}$$

In equations (5) and (6), *w*_*o*_ and *b*_*o*_ represent the weight and bias of the output gate. *o*^*t*^ represents the output gating unit, *h*^*t*^ is the output value of the current unit, and *σ* represents the activation function.

### 2.3. Volleyball-Movement Pose Recognition Method Based on LSTM-Attention

**Figure 3:**
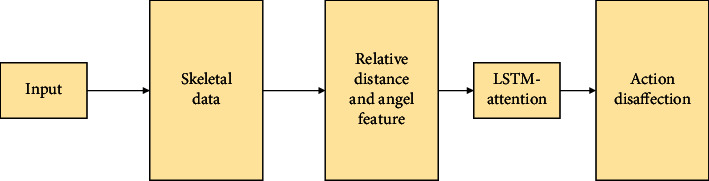
Flowchart of volleyball gesture recognition.

This section constructs a volleyball-movement posture recognition method based on LSTM-Attention to help LSTM effectively extract the feature information before and after the action [16]. This section first gives a brief overview of the model and analyses the various modules involved. The experimental results demonstrate the effectiveness of the LSTM-Attention method proposed here in ball-motion pose recognition. In the recent years, the research on human gesture recognition has gradually replaced the status of traditional methods with the continuous development of DL-related technologies. In human gesture recognition, although the RNN-based gesture recognition method has obvious advantages in short-term memory, it has great difficulties in dealing with some recognition scenarios that require long-term memory. As a special type of the RNN, LSTM can not only solve the problem of the disappearance of the RNN gradient but also enhance the network's ability to memorize information for long time intervals. The latter is favored by many researchers compared with the former because it is good at obtaining feature information between long-term sequences. Many networks optimized based on this have been produced with the in-depth study of the LSTM neural network. They are widely used in text, speech, and image recognition. In the research on human posture recognition, different human skeletal structures will produce great differences when they are playing volleyball. This can lead to indistinguishable target users in the same ball game pose. For this problem, this article proposes a feature enhancement preprocessing method based on the angle and the relative distance. Besides, contextual information between action sequences plays a crucial role in gesture recognition. This section constructs a ball-motion pose recognition method based on the LSTM-Attention model to effectively extract the long-sequence feature information of ball motion poses. The overall process of ball sports gesture recognition is shown in Figure 3.

First, Kinect is used to acquire the joint point coordinate data of the human skeleton performing ball motion [17]. Then, the scale-invariant angle and relative distance features are extracted from the joint point information. Finally, the LSTM-attention network is used to mine the deep timing information in the skeleton sequence of the human body when ball sports are performed. Furthermore, this information is combined with spatial features to recognize the ball motion pose of the human body [18]. The model learns the correlation between the time series data of ball motion poses autonomously and effectively by combining LSTM with the attention mechanism to improve the accuracy of the model pose recognition.

### 2.4. Angle Feature Extraction

In the process of the human body performing ball sports, the features selected based on joint point information should have general behavior [19]. Features do not vary greatly due to differences in the human skeletal structure, and they do not shift because the target user and the Kinect depth sensor are in different positions. Therefore, the angle features extracted based on the joint point information are used to predict the pose category of the human body during ball sports [20]. When the human body performs different ball sports, there will be different angular relationships between the joint points of the human bones in space. Especially for the joint points on the arm, the included angle between the joint points involved in the corresponding action of the ball sports will have a relatively fixed range of variation. This can intuitively describe the ball game posture. Therefore, the coordinate information of the eight joint points is decomposed into five parts according to the human body structure, including the trunk and the limbs. Then, the angle feature extraction is performed on the joint-point coordinate information of these five parts.

When the angle of the joint point information of the human skeleton is calculated, the limbs of each part in the human skeleton model need to be regarded as a vector. The correspondence between the angles between the joint points and each component vector is shown in Table 1. *r*_*ij*_ represents the vector that forms the angle of a joint point.

**Figure 4:**
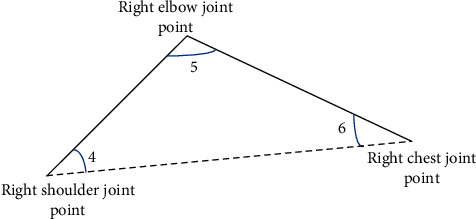
Schematic diagram of the characteristics of the right arm.

In Figure 4, the right arm model of the human body is taken as an example [21], and the joint angle *r*_5_ consists of two vectors, *r*_4,5_ and *r*_5,6_, respectively.

### 2.5. Relative Distance Feature Extraction

The relative distance feature between the human skeleton joint points is a kind of information that can express different ball-motion pose data in the spatial dimension [22]. When the human body performs a ball action, the spatial position information of the joint points of each part of the human skeleton will also change. Moreover, the relative distance between some joint points that change with the movement will also form a change rule for an action posture [23]. For example, when people perform a badminton swing, the relative distance between the user's hand and the base of the spine is a very expressive information. Therefore, the relative distance feature extracted based on joint point information is used here to analyse the pose category of the human body when they perform ball sports.

It is found that the joint points at the base of the spine have stability in the process of the human body movement in expressing the human body ball sports posture through analysis of the joint point data when the human body performs ball sports posture. Therefore, this article regards the joint point at the base of the spine as the center point and analyses the ball sports posture based on the relative distance between the center point and other joint points.

### 2.6. Ball-Movement Pose Recognition Method Based on LSTM-Attention

**Figure 5:**
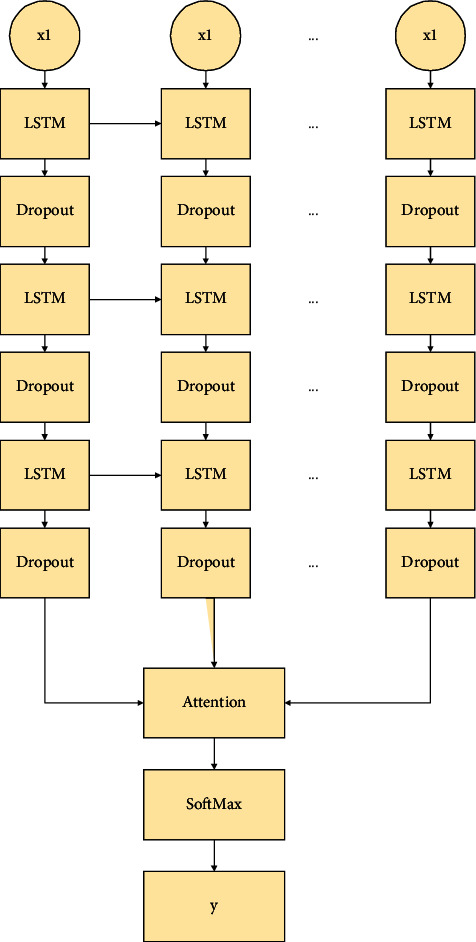
Network structure of volleyball pose recognition.

Here, Kinect is used to collect the joint point data of the human body during ball sports [24]. Based on this, two kinds of geometric features are artificially designed to describe the pose of the ball. The appropriate relevant parameters in the model are obtained after the LSTM-attention network model constructed here is trained on the ball sports training set. Besides, the recognition and classification of ball sports poses are carried out on the test set. Its network structure is demonstrated in Figure 5.

In this model, a multilevel LSTM structure is designed to improve the learning ability of the recognition network [25] to handle complex feature representations in ball sports poses. The number of network layers of LTSM is designed to be three layers. An attention mechanism is added to the model. This design enables the feature vector to spontaneously perceive the network weights that significantly impact the recognition results of ball motion gestures. Some important feature information gets attention. This can also perform further feature enhancement on the feature data extracted from the previous network layers. In addition, a dropout layer is added between the LSTM structures. This can reduce the occurrence of overfitting of the gesture recognition model when the number of experimental samples is limited. To sum up, feature learning through the multilevel LSTM network is combined with feature enhancement of the attention mechanism. This enables the network model to fully and effectively learn the correlation between the time series data in the ball motion poses, thereby improving the pose recognition ability of the entire network model.

Figure 5 displays a diagram of the LSTM-attention network model that is expanded according to time. The model can form an action sequence according to the time sequence of the features of the human body during ball sports, and it is used as the input of the ball-sports pose recognition network. The input includes an angle feature and a relative distance feature. The ball motion features of each human body have become a 38-dimensional data through the previously mentioned joint evaluation, repair, and feature extraction. Also, the length of each action sequence will be affected by the different frame numbers of different actions. It is indispensable to perform isometric operations on the data in the dataset before the feature data are input into the gesture recognition network model. According to the longest frame value in each ball motion sequence, the other motion sequences are zeroed.

The multidimensional feature sequence is input into the LSTM-attention network, which is processed by LSTM, dropout, and the attention mechanism. The intermediate value is sent to the output layer. The function used in this layer is the softmax function. The function can judge the corresponding ball sports posture and output the probability value of five different ball-sports posture labels. Finally, the maximum value of the probability evaluation values is selected as the output category of the final ball-motion pose.

When the human body performs different ball motion poses, all the joint motion data contained in the human skeleton are not equally important. For example, the changes in the joint point data of the human bones are mainly concentrated in the right arm part in the process of the human body completing the badminton swing. The joint point data of other parts of the body have little effect on the final gesture recognition effect. Therefore, the attention mechanism is introduced into the improved human ball-motion pose recognition model. In the process of movement, the important data of human limbs and joints can be marked and much attention can be given to them. In the research data here, the coordinate data of fifteen joint points of the human body during ball sports are collected, and the human skeleton model is established based on this. In most cases, the joint point information that a ball motion pose can be associated with is fixed. These fixed-joint point information will be converted into feature vectors through LSTM. The essence of the attention mechanism is to perform a weighted summation of these feature vectors to find out the joint point information that importantly impacts the recognition of ball motion poses.

## 3. Results and Discussion

**Figure 6:**
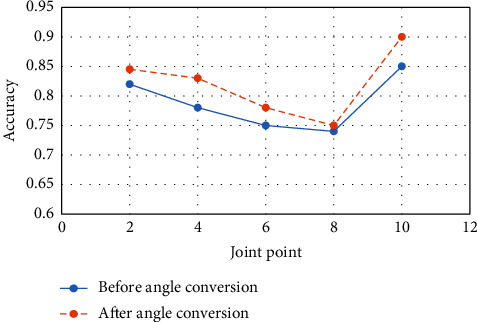
LSTM prediction results before and after angle conversion.

The comparative experiments before and after the coordinate system transformation of joint point information verify the improvement effect of the joint-point preprocessing method based on the coordinate system transformation on the accuracy of gesture recognition. This section conducts experiments. First, this article conducts experiments on the joint point data before and after the coordinate system transformation through the traditional LSTM-pose recognition network model. MATLAB software is used to simulate and simulate the gesture recognition process of the network model. The results are shown in Figure 6.

Figure 6 shows the comparative experimental results before and after the coordinate information conversion preprocessing of the joint point data through the LSTM neural network. The experimental results indicate that the joint-point coordinate information of the coordinate system transformation significantly improves the recognition accuracy of the five ball motion poses by at least 0.01. However, the improvement in gesture recognition accuracy for smash actions is lower compared to the improvements in gesture recognition accuracy for serve, lift, high clear, and backhand. This also means that gesture recognition accuracy for actions like smash is less affected by angular changes during data collection than other actions.

**Figure 7:**
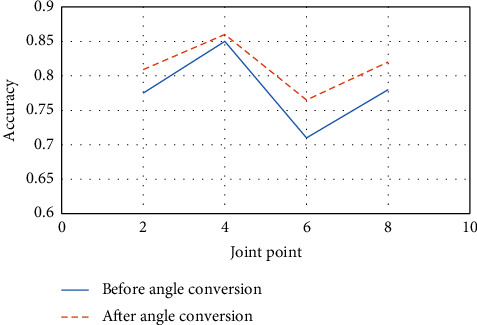
Overall prediction results before and after angle conversion.

In addition to LSTM, this section also conducts comparative experiments on gesture recognition methods such as BiLSTM, linear SVM, and multilayer LSTM. The experimental data and settings remain the same as in the above LSTM experiments. BiLSTM is the abbreviation of bidirectional long short-term memory, which means a bidirectional long and short-term neural network. It is composed of forward LSTM and backward LSTM. Both are often used to model contextual information in natural language processing tasks.  Figure 7 shows the overall prediction results of each gesture recognition method for the joint-point coordinate information before and after the coordinate system conversion.

The experimental results show that the joint-point coordinate information after the coordinate system transformation can effectively improve the overall accuracy of the ball-motion gesture recognition network model. After converting the coordinate system, the accuracy is improved by at least 0.01, and the accuracy of the method proposed here is higher than the other methods. The optimal hierarchical experiment of the LSTM multilayer structure is expected to integrate the feature information of the long-term sequence on a global scale and realize the high-level abstraction of the input human skeleton joint point data. Therefore, this article constructs a multilevel LSTM structure based on a classification model. However, if the number of layers in the LSTM multilayer structure is large, the model will take a lot of time to converge, which will complicate the model. Therefore, this article conducts comparative experiments on different layers of LSTM structures on the BadmintonData and MSRAction3D datasets to verify the scientificity and effectiveness of the ball-motion gesture recognition model based on the three-layer LSTM structure designed here. The BadmintonData dataset contains various volleyball poses. The MSRAction3D dataset records 20 actions and ten subjects. Each subject performs each action two to three times. There are a total of 567 depth map sequences with a resolution of 640 *∗* 240. Data are recorded with a depth sensor similar to the Kinect unit.

**Figure 8:**
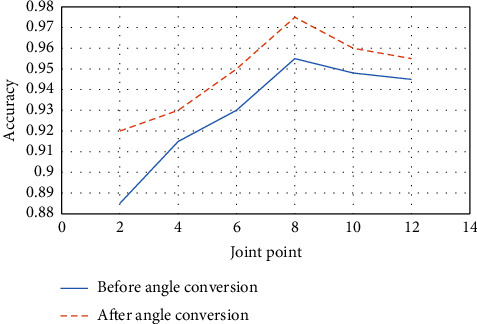
Comparative experiments of different LSTM layer structures in action recognition.

The number of layers of the LSTM multilayer structure is set as one, two, three, four, and five, respectively. LSTM-Attention_*n* is a model that fuses the *n*-layer LSTM structure and the attention mechanism, respectively. Besides, LSTM is a model that only contains a single-layer LSTM structure without an attention mechanism. The results are revealed in Figure 8.

The purpose of the comparative experiment before and after the restoration of joint point information is to verify the improvement effect of the joint-point processing method based on the bone length and motion continuity on the performance of the pose recognition method. Its essence is to evaluate the influence of the joint point data with errors on the accuracy of human pose recognition.

This section uses the abovementioned gesture recognition methods to conduct comparative experiments on the BadmintonData and MSRAction3D datasets. In the experiment, other experimental configurations are the same except for the preprocessing process of joint-point data repair. The two datasets after joint data repair processing are recorded as BadmintonData_Repairing and MSRAction3D_Repairing, respectively. Then, model generation and prediction are performed on the BadmintonData, MSRAction3D, BadmintonData_Repairing, and MSRAction3D_Repairing datasets using the above four pose recognition methods, respectively. The predicted results are shown in Table 2.

From the experiment, the same action poses in the BadmintonData_Repairing and MSRAction3D_Repairing datasets are recognized, and the pose recognition results after the joint repair operation will be much higher than the data without joint repair. It is proved that the processing method based on the bone length and motion continuity proposed in this article can improve the final accuracy of the gesture recognition network. Therefore, it is crucial to evaluate the reliability of the joint-point coordinate information obtained by Kinect and restore the joint point information with errors before the joint-point coordinate information is used for action and pose recognition.

## 4. Conclusion

Studying, recognizing, and detecting volleyball postures can provide theoretical guidance and suggestions for people, which can be applied in competitions. It also helps event judges make sound decisions. At present, the pose recognition of ball sports is very challenging in action complexity and research data, and this research also has an important application value. This article takes volleyball as the representative to study the problem of volleyball movement pose recognition combined with the analysis and summary of the existing human pose recognition research based on the joint point sequence and the LSTM network. This article proposes a data preprocessing method based on the angle and relative distance feature enhancement and a volleyball motion pose recognition model based on LSTM-attention. The experimental results imply that the data preprocessing method reported here can further improve the accuracy of gesture recognition. The joint-point coordinate information of the coordinate system transformation significantly improves the recognition accuracy of the five ball motion poses by at least 0.01. Moreover, it is concluded that the LSTM-attention recognition model is not only scientific in structure design but also has considerable competitiveness in gesture recognition performance. However, this method is not yet universal and should be refined in future research.

## Figures and Tables

**Table 1:** Correspondence between the angle between the joint points and each component vector.

Joint angle number	Composition vector
1	*r*_1,2_, *r*_1,3_
2	*r*_2,1_, *r*_2,3_
3	*r*_3,1_, *r*_3,2_
4	*r*_4,5_, *r*_4,6_
5	*r*_5,4_, *r*_5,6_
6	*r*_6,4_, *r*_6,5_
7	*r*_7,8_, *r*_7,9_
8	*r*_8,7_, *r*_8,9_

**Table 2:** Comparative experiments before and after the data recovery of joint points.

Experimental data	LSTM _	B I-LSTM	Linear SVM	Multilayer LSTM
Badminton data	0.81	0.87	0.76	0.82
BadmintonData_Repairing	0.83	0.88	0.83	0.85
MSRAction3D_	0.90	0.95	0.92	0.95
MSRAction3D_Repairing	0.92	0.96	0.93	0.95

## Data Availability

The experimental data used to support the findings of this study are available from the corresponding author upon request.
